# Supplementary material for: Synergistic antibacterial effect of hydroxyl radicals generated by the combination of hypochlorous acid and UV irradiation
Source: PLoS One. 2025 Oct 15;20(10):e0334003. doi: 10.1371/journal.pone.0334003 (PMC12527167; doi:10.1371/journal.pone.0334003)
Supplement: Attachment — Submitted filename: response to Reviewers.docx [file pone.0334003.s002.docx]

Dear reviewers

I appreciate the kind comments.

Here are the answers to those comments.

Reviewer #1:

The submitted manuscript is original and proposes solutions to problems that lack new technologies to solve them. However, the authors do not consider the impact that the application of their proposal based on UVC/HOCl processes could have on the formation of toxic chlorinated species that could be introduced into living beings, causing a greater problem. I think the authors should expand their study by addressing the byproducts that are being produced, especially because they highlight the effect of organic matter in the process and ignore the generation of chlorinated byproducts. Additionally, the authors should modify the introduction to address more the state of the art of the oxidative radicals of the radiation/chlorine system in the inactivation of pathogens, if the novelty of the study lies in the role of hydroxyl radicals in the inactivation of bacteria.

Answer : I agree with the effect of byproducts produced by UVC/HOCl process. The process produces OH radical which can affect living organisms and kill viruses and bacteria that we call as disinfection. The production and effect of chlorinated byproducts was mentioned in “Introduction” section and previous studies were cited for potential use of the process. In addition, the result was discussed with previous studies which showed adverse effect on living organisms.

Reviewer #2:

1. More findings of the work should be added to the abstract.

Answer : Abstract was revised to address more findings of this study.

2.Please check and complete conditions and procedures in the experimental section and all procedures should be written clearly in detail.

Answer : The experimental procedure and test conditions were clearly described in the experimental section.

3.Please clarify the innovations of this work in the introduction section.

Answer : The meaning of this study and aims were more clearly mentioned to clarify the innovation of the study.

4.Include the purity of the used chemicals in the text.

Answer : The purity of the chemicals used is included in the text.

5.Discuss the effect of scavenger to confirm the role of OH free radical and other reactive species.

Answer : To investigate the effect of radical consumption and the reduction in disinfection effect due to organic substances in the air, yeast was used as a scavenger for the radical effect. The phenomenon of a decrease in the disinfection effect of Salmonella bacteria when airborne yeast is present was observed, and this result was examined by comparing it with the scavenger role suggested in previous research papers and revised in result and discussion section.

6.The authors need to provide the standard antibiotics details in the antibacterial activity assay.

Answer : In this study the standard antibiotics as the positive control was not used. We investigated the hindrance of organic material

7.Deeply check and polish English language level, there are some spelling and grammatical errors.

Answer : English expressions are reviewed from the native speaker.
